# Exploring gender differences in the relationship between gut microbiome and depression - a scoping review

**DOI:** 10.3389/fpsyt.2024.1361145

**Published:** 2024-02-19

**Authors:** Leila Niemela, Gillian Lamoury, Susan Carroll, Marita Morgia, Albert Yeung, Byeongsang Oh

**Affiliations:** ^1^ Sydney Medical School, University of Sydney, Sydney, NSW, Australia; ^2^ Northern Sydney Cancer Centre, Royal North Shore Hospital, Sydney, NSW, Australia; ^3^ Massachusetts General Hospital, Harvard Medical School, Boston, MA, United States

**Keywords:** gut microbiome, depression, gender, biomarker, gut dysbiosis

## Abstract

**Background:**

Major depressive disorder (MDD) exhibits gender disparities, and emerging evidence suggests the involvement of the gut microbiome, necessitating exploration of sex-specific differences.

**Methods:**

A review was conducted, encompassing a thorough examination of relevant studies available in Medline via Ovid, Embase via OvidSP, CINAHL, and PsycINFO databases from their inception to June 2023. The search strategy employed specific keywords and Medical Subject Headings (MeSH) terms tailored to major depressive disorder in women, encompassing unipolar depression, depressive symptoms, and dysbiosis.

**Results:**

Five studies were included. Among the four studies, alterations in alpha (n=1) and beta diversity (n=3) in the gut microbiome of individuals with MDD were revealed compared to controls. Gender-specific differences were observed in four studies, demonstrating the abundance of specific bacterial taxa and highlighting potential sex-specific implications in MDD pathophysiology. Correlation analyses (n=4) indicated associations between certain bacterial taxa and the severity of depressive symptoms, with varying patterns between males and females. Studies (n=3) also highlighted promising findings regarding the potential utility of microbial markers in diagnosing MDD, emphasizing the crucial role of sex stratification in understanding the disease pathophysiology.

**Conclusions:**

The findings underscore the importance of recognizing gender-specific differences in the composition of the gut microbiome and its relationship with MDD. Further comprehensive robust studies are required to unravel the intricate mechanisms underlying these disparities.

## Introduction

Major Depression, also known as major depressive disorder (MDD), is a prevalent mental and emotional ailment affecting an estimated 185 million people globally (1). The World Health Organization classified depression as the fourth-leading burden of disease globally in 2008, with projections indicating it could become the second-leading cause by 2030 (2). Women are disproportionately affected, experiencing nearly double the prevalence compared to men (1), a trend observed across both developed and developing countries (3).

Various theories such as the biopsychosocial model, have attempted to elucidate the underlying reasons for this gender disparity, pointing to differences in hormones (4, 5), neurotransmitters (5, 6), and brain structure (7, 8). Recent research has also explored the intricate relationship between the gut microbiome and depression, uncovering potential links through the gut-brain axis (9–31). While significant advancements have been made, there remains a dearth of evidence to precisely elucidate the mechanisms driving these disparities or the potential for sex-specific biomarkers.

The concept of ‘gut dysbiosis’ - an abnormal alteration in the composition and function of the gut microbiome - has gained traction as a potential player in the pathogenesis of MDD and other psychiatric disorders (9–31). The intricate communication between the gut microbiome and the brain through various pathways, including neural, immune, and metabolic mechanisms, presents a promising avenue for further exploration. Recent studies have highlighted differences in the gut microbiota composition between individuals with MDD and control groups, pointing to potential sex-specific differences that warrant further investigation (19, 32–35).

This scoping review aims to explore the existing evidence on the relationship between major depression and the gut microbiome, particularly in the context of women, while also summarizing the sex-specific differences in the gut microbiome profiles of male and female subjects with major depression.

## Methods

A comprehensive literature search was conducted from database inception to June 2023 in Medline via Ovid (1946-present), Embase via OvidSP (1947-present), Cinahl Complete, and PsycINFO via Ovid (1806-present). The search used specific keywords and MeSH terms related to major depression in women, including unipolar depression, depressive symptoms, and dysbiosis.

Inclusion criteria encompassed studies with adult human participants of both sexes, focusing on female-specific outcomes. Studies investigating the relationship between major depression and gastrointestinal microbiota in adult humans were included, while those exclusively concerning other psychiatric disorders (e.g., schizophrenia, chronic stress, PTSD, bipolar disorder), subtypes of depression (e.g., postpartum, late-life depression), or other medical conditions were excluded. Additionally, studies involving females under 18 years old were not considered.

## Results

**Figure 1:**
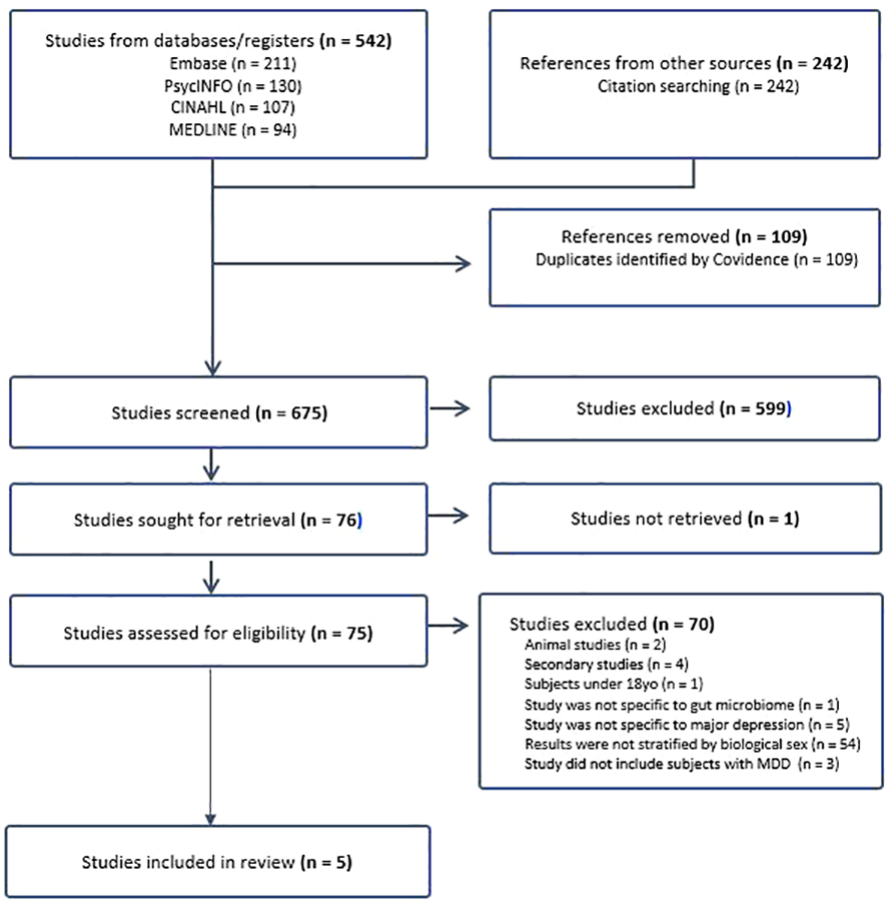
Flow chart.

From the initial database search, 784 studies were identified, and after removing 109 duplicates, 675 studies underwent phase one screening. Following this, 76 studies were subjected to full-text retrieval, resulting in 75 fully assessed articles. Ultimately, five articles were included in the literature review (**Figure 1** for the PRISMA flow chart).

### Characteristics of studies

The review included a total of (n=780) subjects from case-controlled studies in China and (n=1104) subjects from a retrospective cohort study in Germany. Among the case-control studies, (n=239) female and (n=125) male subjects with MDD were compared to (n=261) female and (n=155) male healthy controls. Notably, one study by Li et al. (33) involved subjects with Bipolar disorder (BD) (n=166) experiencing a depressive episode, whose data were excluded from this review’s analysis (**Table 1.1**).

**Table 1.1:** Characteristics of studies.

	Participant details					Recruitment location	Assessment tool	Sample analysis
	Female (n)	Male (n)	Average age	Subjects with MDD or DS	Medication status			
Chen et al 2018, China Case Control Study (32)	MDD (n=24) HC (n=24)	MDD (n=20) HC (n=20)	MDD (F, M) 42 yrs, 40 yrs HC (F, M) 44 yrs, 43 yrs	MDD patients undergoing first episode MDD	Drug naive	MDD in hospital HC in community	HDRS-17	16S rRNA
Li et al 2022, China Case Control Study (33)	MDD (n=77) HC (n=100) BD (n=83)	MDD (n=43) HC (n=71) BD (n=82)	MDD (F, M) 26 yrs, 26 yrs HC (F, M) 27 yrs, 26 yrs	MDD patients undergoing depressive episode	Unmedicated	MDD in hospital HC in community	DSM-IV HAMD	16S rRNA
Chen et al 2021, China Case Control Study (34)	MDD (n=62) HC (n=46)	Nil	MDD (F): 40 yrs HC (F): 37 yrs	MDD patients with HAMD-17 score ≥ 18	Medicated (n= 26) Unmedicated (n= 36)	MDD in hospital	DSM-IV HAMD-17	16S rRNA and shotgun metagenomic sequencing
Hu et al 2023, China Cross sectional study (35)	MDD (n=76) HC (n=91)	MDD (n=62) HC (n=64)	MDD: 29 yrs HC: 29 yrs	MDD patients	Unmedicated	MDD in hospital HC in community	DSM-IV HAMD-17	Shotgun metagenome sequencing
Chung et al 2022, Germany Retrospective Cohort Study (19)	DS (n=339) HC (n=339)	DS (n=213) HC (n=213)	DS:50 yrs HC:50 yrs	Adults in community with clinical diagnosis of dysbiosis	Unmedicated	DS in community HC in community	ICD-10	Clinical record of dysbiosis

MDD, Major depressive disorder; HC, Healthy Control; BD, Bipolar Disorder; DS, Dysbiosis; F, Female; M, Male; DSM, Diagnostic and Statistical Manual of Mental Disorders (-Text revision); HAMD or HDRS, Hamilton Depression Rating Scale (-Text revision); NR, Not reported; 26 patients had used antidepressants for less than 3 consecutive days in 2 weeks prior to faecal collection.^2^ Adults (≥18 yrs) ≥ 1 visit to general practitioner; and ≥1 diagnosis of dysbiosis ≥ 3 months after initial diagnosis.

### Gender-specific microbiome diversity alterations in subjects with major depression

Alpha diversity remained unchanged in MDD subjects across three studies (32–34), while one study (35) reported a reduction. Beta diversity analysis revealed significant differences in both male and female MDD groups compared to matched healthy controls (HCs) in studies by Chen and Li (32, 33). In the female-only study by Chen et al. (34), alterations in beta diversity were observed only at the species level in female MDD subjects. Notably, Li et al. (33) found that while alpha diversity was significantly higher in female healthy controls compared to male healthy controls, this difference was not evident in the context of depression. **Table 1.2** provides an overview of the key findings.

**Table 1.2:** Gender-Specific Microbiome Profile Alterations in Subjects with Major Depression.

Diversity	Alpha Diversity				Beta Diversity	
	MDD vs HC	MDD vs HC		MDD	MDD vs HC	
	Female	Male		Female vs Male	Female	Male
Chen et al., 2018 (32)	NS	NS		NS	*	*
Li et al., 2022 (33)	NS	NS		**---**	*	*
Chen et al 2021 (34)	NS	NS		N/A	NS (16SrRNA) *(SMG)	N/A
Hu et al., 2023 (35)	*↓	*↓		**---**	**---**	**---**

MDD, Major Depressive Disorder; HC, Healthy Controls; NS, No significant difference; *Significant difference; **---**, not reported; *↓Significantly decreased; N/A, not applicable; 16s, 16S rRNA gene sequencing; SMG, shotgun metagenomic sequencing.

### Gender-specific microbiome profile alterations in subjects with major depression

All case-control studies (32–34), highlighted notable differences in gut microbiota between individuals with major depressive disorder (MDD) and the respective control groups. These distinctions were particularly pronounced when comparing male and female cohorts. Further details can be found in **Table 1.3**. Upon examining studies encompassing both male and female subjects, females with MDD exhibited a higher relative abundance of *Actinobacteria, Firmicutes, and Bacteroidetes* compared to the control group (32, 33). In male MDD patients, an increase and decrease in *Bacteroidetes* clusters, along with an increase in *Firmicutes* clusters, was observed. In the study conducted by Chen et al. (34) focusing on female MDD patients, an increase in Bacteroidetes, Proteobacteria, Fusobacteria, and *Verruomicrobia*, and a decrease *in Firmicutes and Actinobacteria* was reported. Notably, only two studies (34, 35) investigated the microbiome at the species level, revealing significant changes at the family, genus, and species levels.

**Table 1.3:** Gender-Specific Microbiome Profile Alterations in Subjects with MDD compared to healthy controls.

	Phylum		Family		Genus		Species	
	Female	Male	Female	Male	Female	Male	Female	Male
Chen et al., 2018 (32)	Actinobacteria **↑** Actinobacteria **↑**	Bacteroidetes **↓** Bacteroidetes **↑**	Coriobacteriaceae ↑ Lachnospiraceae ↑ Ruminococcaceae ↑ Lachnospiraceae ↓ Ruminococcaceae ↓	Erysipelotrichaceae ↑ Lachnospiraceae ↑ Lachnospiraceae ↓ Ruminococcaceae ↓	*Actinomyces* ↑ *Bifidobacterium* ↑ *Asaccharobacter* ↑ *Atopobium* ↑ *Eggerthella* ↑ *Gordonibacter* ↑ *Olsenella* ↑ *Eubacterium* ↑ *Anaerostipes* ↑ *Blautia* ↑ *Roseburia* ↑ *Faecali-bacterium* ↑ *Desulfovibrio* ↑ *Howardella* ↓ *Sutterella* ↓ *Pyramidobacter* ↓	Bacteroides ↑ Erysipelotrichaceae incertae sedis ↑ Veillonella ↑ Atopobium ↑ *Anaerovorax* ↓ *Gordonibacter* ↓ *Pyramidobacter* ↓	NR	NR
Li et al., 2022 (33)	Firmicutes **↑** Bacteroidetes **↑**	Firmicutes **↑**	Lachnospiraceae **↑** Bacteroidaceae **↑** Bacteroidaceae **↑** Bacteroidaceae **↑** Bacteroidaceae **↑**	Lachnospiraceae **↑**	NR	NR	NR	NR
Chen et al 2021 (34)	16s: Bacteroidetes **↑** Proteobaceteria **↑** Fusobacteria **↑** Firmicutes **↓** Actinobacteria **↓** SMG: Bacteroidetes **↑** Verrucomicrobia **↑** Fusobacteria **↑** Firmicutes **↓**	NA	*Enterobacteriaceae* **↑** *Tannerellaceae* **↑** *Burkholderiaceae* **↑** *Campylobacteraceae* **↑** *Corynebacteriaceae* **↑** *Clostridia_unclassified* **↑** *Ruminococcaceae* **↓** *Lachnospiraceae* **↓** *Coriobacteriales_unclassified* **↓**	NA	Escherichia-Shigella **↑** Prevotellaceae_NK3B31_group **↑** Hungatella **↑** Campylobacter **↑** Raoultella **↑** Barnesiella **↑** Coprobacillus **↑** Clostridium_innocuum_group **↑** Alistipes **↑** Enterobacteriaceae_unclassified **↑** Lachnoclostridium **↑** Prevotellaceae_unclassified **↑** Flavonifractor **↑** Eisenbergiella **↑** Anaerotruncus **↑** Anaeroglobus **↑** Mobiluncus **↑** Rodentibacter **↑** Fastidiosipila **↑** Finegoldia **↑** Aerococcus **↑** Ruminococcaceae_uncultured **↑** Turicibacter **↑** S5-A14a **↑** Parabacteroides **↑** GCA-900066755 **↑** Clostridia_unclassified **↑** Morganella **↑** Agathobacter **↓** Butyricicoccus **↓** Faecalibacterium **↓** Dorea **↓** Coprococcus_3 **↓** Ruminococcaceae_UCG-013 **↓** Eubacterium_ventriosum_group **↓** Lachnospiraceae_FCS020_group **↓** Eubacterium_hallii_group **↓** Blautia **↓** Anaerostipes **↓** Lachnospiraceae_NK4A136_group **↓** Lachnospiraceae_UCG-001 **↓** Erysipelotrichaceae_UCG-003 **↓** Coprococcus_1 **↓** Subdoligranulum **↓** Tyzzerella_3 **↓** CAG-56 **↓** Lachnospiraceae_ND3007_group **↓** Coriobacteriales_unclassified **↓** Moraxellaceae_unclassified **↓** Ruminococcus_1 **↓** Roseburia **↓** Ruminiclostridium **↓** Ruminococcus_2 **↓** Alcaligenes **↓** Fusicatenibacter **↓** Lachnospiraceae_UCG-006 **↓** Burkholderia-Caballeronia-Paraburkholderia **↓** Candidatus_Saccharimonas **↓** F0332 **↓** Bifidobacterium **↓** *SMG:* Granulicella **↑** Adlercreutzia **↑** Barnesiella **↑** Parabacteroides **↑** Paraprevotella **↑** Alistipes **↑** Clostridiales_noname **↑** Flavonifractor **↑** Oscillibacter **↑** Anaerotruncus **↑** Ruminococcaceae_noname **↑** Bilophila **↑** Campylobacter **↑** Akkermansia **↑** Gammaretrovirus **↑** Lactobacillus **↓** Eubacterium **↓** Dorea **↓** Roseburia **↓** Faecalibacterium **↓** Megamonas **↓** Megasphaera **↓** Haemophilus **↓**	NA	Clostridium_asparagiforme **↑** Alistipes_onderdonkii **↑** Clostridium_citroniae **↑** Barnesiella_intestinihominis **↑** Alistipes_finegoldii **↑** Oscillibacter_unclassified **↑** Clostridium_hathewayi **↑** Clostridiales_bacterium_1_7_47FAA **↑** Flavonifractor_plautii **↑** Clostridium_bolteae **↑** Akkermansia_muciniphila **↑** Porphyromonas_uenonis **↑** Campylobacter_hominis **↑** Adlercreutzia_equolifaciens **↑** Lachnospiraceae_bacterium_7_1_58FAA **↑** Murine_osteosarcoma_virus **↑** Anaerotruncus_unclassified **↑** Bilophila_wadsworthia **↑** Porphyromonas_asaccharolytica **↑** Erysipelotrichaceae_bacterium_2_2_44A **↑** Bacteroides_caccae **↑** Bilophila_unclassified **↑** Granulicella_unclassified **↑** Atopobium_vaginae **↑** Paraprevotella_unclassified **↑** Paraprevotella_xylaniphila **↑** Ruminococcaceae_bacterium_D16 **↑** Subdoligranulum_sp_4_3_54A2FAA **↑** Erysipelotrichaceae_bacterium_21_3 **↑** Campylobacter_ureolyticus **↑** Megamonas_unclassified **↓** Faecalibacterium_prausnitzii **↓** Eubacterium_rectale **↓** Haemophilus_parainfluenzae **↓** Dorea_longicatena **↓** Roseburia_hominis **↓** Roseburia_inulinivorans **↓** Megamonas_hypermegale **↓** Bacteroides_plebeius **↓** Streptococcus_australis **↓** Weissella_cibaria **↓** Megamonas_funiformis **↓** Megasphaera_unclassified **↓** Bacteroides_xylanisolvens **↓** Streptococcus_salivarius **↓**	NR
Hu et al., 2023 (35)	NR	NR	Bacteroidaceae **↑** Prevotellaceae **↑** Bifidobacteriaceae **↑** *Ruminococcaceae* **↓** Lachnospiraceae **↓** Enterobacteriaceae **↓** Eubacteriaceae **↓**	Bacteroidaceae **↑** Prevotellaceae **↑** Bifidobacteriaceae **↑** Ruminococcaceae **↓** Lachnospiraceae **↓** Enterobacteriaceae **↓** Eubacteriaceae **↓** Clostridiaceae **↓** Veillonellaceae **↓**	Bacteroides **↑** Butyricimonas **↑** Faecalibacterium **↑** Clostridium **↑** Ruminiclostridium **↑** Parabacteroides **↑** Clostridium **↓** Roseburia **↓** Faecalibacterium **↓** Eubacterium **↓** Blautia **↓** Dorea **↓** Anaerostipes **↓** Akkermansia **↓** Ruminococcus **↓** Subdoligranulum **↓** Klebsiella **↓** unclassified_p:Firmicutes **↓**	Bacteroides **↑** Blautia **↑** Bilophila **↑** Clostridium **↑** Eubacterium **↑** Parabacteroides **↑** Parasutterella **↑** Phascolarctobacterium **↑** unclassified_p:Proteobacteria **↑** Sutterella **↑** Eubacterium **↓** Faecalibacterium **↓** Adlercreutzia **↓** Anaerostipes **↓** Blautia **↓** Citrobacter**↓** Clostridium **↓** Coprococcus **↓** Dialister **↓** Dorea **↓** Enterobacter **↓** Enterococcus **↓** unclassified_p:Firmicutes **↓** Klebsiella **↓** Lactococcus **↓** unclassified_f:Peptostreptococcaceae **↓** Ruminococcus **↓** Salmonella **↓** Subdoligranulum **↓**	Bacteroides_vulgatus **↑** Bacteroides_salyersiae **↑** Bacteroides_stercoris **↑** Bacteroides_thetaiotaomicron **↑** Bacteroides_massiliensis **↑** Bacteroides_stercoris_CAG:120 **↑** Bacteroides_dorei **↑** Bacteroides_fragilis **↑** Bacteroides_sp._3_1_33FAA **↑** Bacteroides_sp._CAG:98 **↑** Bacteroides_ovatus **↑** Butyricimonas_virosa **↑** Eubacterium_siraeum **↑** Parabacteroides_distasonis **↑** Clostridium_sp._CAG:7 **↑** Clostridium_sp._CAG:217 **↓** Roseburia_intestinalis **↓** Faecalibacterium_prausnitzii **↓** Clostridium_sp._CAG:510 **↓** Faecalibacterium_sp._CAG:82 **↓** Eubacterium_ventriosum **↓** Blautia_obeum **↓** Blautia_wexlerae **↓** Blautia_sp._Marseille-P2398 **↓** Eubacterium_hallii **↓** Dorea_formicigenerans **↓** Anaerostipes_hadrus **↓** Eubacterium_hallii_CAG:12 **↓** Eubacterium_sp._CAG:202 **↓** Akkermansia_muciniphila_CAG:154 **↓** Ruminococcus_sp._5_1_39BFAA **↓** Eubacterium_sp._CAG:156 **↓** Clostridium_sp._CAG:417 **↓** Dorea_longicatena **↓** Subdoligranulum_variabile **↓** Klebsiella_pneumoniae **↓** Eubacterium_sp._CAG:115 **↓** Firmicutes_bacterium_CAG:41 **↓**	Ruminococcus_gnavus **↑** Bacteroides_caccae **↑** Bacteroides_dorei **↑** Bacteroides_eggerthii **↑** Bacteroides_finegoldii **↑** Bacteroides_fragilis **↑** Bacteroides_massiliensis **↑** Bacteroides_ovatus **↑** Bacteroides_sp._3_1_33FAA **↑** Bacteroides_sp._3_1_40A **↑** Bacteroides_sp._4_3_47FAA **↑** Bacteroides_sp._9_1_42FAA **↑** Bacteroides_sp._CAG:98 **↑** Bacteroides_stercoris **↑** Bacteroides_thetaiotaomicron **↑** Bacteroides_uniformis **↑** Bacteroides_vulgatus **↑** Bacteroides_xylanisolvens **↑** Bilophila_wadsworthia **↑** Clostridium_sp._CAG:81 **↑** Coprobacillus_sp._CAG:235 **↑** Eubacterium_sp._CAG:146 **↑** Parabacteroides_distasonis **↑** Parabacteroides_merdae **↑** Parasutterella_excrementihominis **↑** Phascolarctobacterium_sp._CAG:207 **↑** Proteobacteria_bacterium_CAG:139 **↑** Sutterella_wadsworthensis **↑** Eubacterium_hallii **↓** Adlercreutzia_equolifaciens **↓** Anaerostipes_hadrus **↓** Blautia_obeum **↓** Blautia_sp._CAG:237 **↓** Blautia_sp._GD8 **↓** Blautia_sp._KLE_1732 **↓** Blautia_sp._Marseille-P2398 **↓** Blautia_wexlerae **↓** Citrobacter_freundii **↓** Clostridium_dakarense **↓** Clostridium_sp._CAG:62 **↓** Clostridium_sp._CAG:75 **↓** Coprococcus_eutactus **↓** Coprococcus_sp._ART55/1 **↓** Coprococcus_sp._CAG:131 **↓** Dialister_invisus **↓** Dialister_succinatiphilus **↓** Dorea_sp._CAG:105 **↓** Enterobacter_cloacae **↓** Enterobacter_sp._GN02315 **↓** Enterococcus_faecalis **↓** Eubacterium_hallii_CAG:12 **↓** Eubacterium_sp._CAG:115 **↓** Eubacterium_sp._CAG:180 **↓** Eubacterium_sp._CAG:202 **↓** Eubacterium_sp._CAG:251 **↓** Faecalibacterium_sp._CAG:74 **↓** Faecalibacterium_sp._CAG:82 **↓** Firmicutes_bacterium_CAG:227 **↓** Firmicutes_bacterium_CAG:341 **↓** Klebsiella_pneumoniae **↓** actococcus_garvieae **↓** Peptostreptococcaceae_bacterium_VA2 **↓** Ruminococcus_sp._5_1_39BFAA **↓** Ruminococcus_sp._CAG:17 **↓** Ruminococcus_sp._CAG:9 **↓** Ruminococcus_sp._JC304 **↓** Salmonella_enterica **↓** Subdoligranulum_variabile **↓**

**↑**,relatively more abundant in subjects with MDD compared to HC; **↓**, relatively less abundant in subjects with MDD compared to HC.

NR, Not reported; NA, Not applicable.

### Correlation of bacterial taxa with severity of depression symptoms

Four studies examined the relationship between the severity of depression symptoms and specific bacterial taxa at the genus level (32–35). Among female MDD subjects, three genera (*Anaerotruncus, Parabacteroides, and Anaeroglobus*) exhibited associations with increased depressive symptoms, whereas five genera (*Clostridium XIVa, Erysipelotrichaceae incertae sedis, Streptococcus, Romboutsia, and Fusicatenibacter*) were linked to reduced depressive symptoms. In male MDD subjects, two distinct genera (*Collinsella, Veillonella*) were found to be correlated with depression symptoms (refer to **Table 1.4**).

**Table 1.4:** Correlation of Bacterial Taxa with Severity of Depression Symptoms.

	Positive Correlation				Negative Correlation				
	Females	Males	All		Females	Males	All		
Chen et al., 2018 (32)		Collinsella	N/A		Clostridium XIVa, Erysipelotrichaceae incertae sedis, Streptococcus	Veillonella	NA		
Li et al., 2022 (33)		NC	N/A		Romboutsia	NC	NA		
Chen et al 2021 (34)	Anaerotruncus, Parabacteroides, Anaeroglobus	NA	N/A		Fusicatenibacter	NA	NA		
Hu et al., 2023 (35)	N/A	NA	Moderate5: Bacteroides	Severe6: Bacteroides	NA	NA	Moderate5: Faecalibacterium Escherichia	Severe6: Ruminococcus Eubacterium

MDD, Major Depressive Disorder; NC, No correlation found; NA, Not assessed. ^5^The severity of MDD was staged with the HAMD-17 scale, moderate depression (score, 17–23), ^6^ The severity of MDD was staged with the HAMD-17 scale, severe depression (score, ≥24).

### Potential diagnostic role of microbial markers and dysbiosis in major depression

Two studies (33, 34) examined the accuracy of microbial markers in diagnosing MDD, identified sex-specific gut microbiota signatures, and evaluated diagnostic performance using the area under the receiver operating characteristic curve (AUC). Analysis of the diagnostic performance sensitivity of these microbial signatures showed area under the curve (AUC) values ranging from 0.79 to 0.92 for females and 0.79 for males with MDD. An additional study (19) investigated the risk of developing MDD within five years following an initial dysbiosis diagnosis and found a stronger association between dysbiosis and MDD diagnosis in males (HR:3.54, 95% CI: 1.75–7.14) compared to females HR:2.61 (95% CI: 1.74 – 3.92). (Refer to **Table 1.5**).

**Table 1.5:** Diagnostic performance of microbial markers and dysbiosis in diagnosis of MDD.

		Diagnostic Performance Sensitivity (AUC)		Microbial Makers			Hazard Ratio7	
				OTU (n)	OTU (n)	Species (n)		
		Female	Male	Female	Male	Female	Female	Male
Li et al., 2022 (33)	16S rRNA	0.795	0.798	11	50	NA	NA	NA
Chen et al 2021 (34)	16S rRNA & Shotgun metagenomic	0.92 (95% CI: 85.3% - 98.8%)	NA	18	NA	45	NA	NA
Chung et al., 2022 (19)	Clinical record 8	NA	NA	NA	NA	NA	2.61 (95% CI: 1.74 – 3.92)	3.54 (95% CI: 1.75–7.14)

AUC, Area under the curve; OTU, Operational taxonomic units; MDD, Major Depressive Disorder; HC, Healthy Controls; CI, Confidence Interval; NA, Not assessed; 7 Hazards Ratio, risk of being diagnosed with depression within five years of dysbiosis.

^8^Clinical Record: Diagnosis of Dysbiosis and MDD (ICD-10 code) recorded in patient clinical record.

## Discussion

Several recent studies have suggested that the gut microbiome profile is associated with Major Depressive Disorder (MDD), yet only a few have investigated the sex-specific link between MDD and the gut microbiome. This review represents the first comprehensive analysis examining the relationship between the gender-specific gut microbiome profile and MDD. To date, five primary studies have provided insights into the relationship between the gut microbiome and MDD in women (19, 32–35). These findings indicate a close association between the gut microbiome composition of females with MDD and the disorder itself, highlighting sex-specific differences in the gut microbiota of MDD patients. Certain genera were found to correlate with the severity of depression, and these correlations varied between males and females. Additionally, sex-specific differences were observed in the diagnostic performance of microbial markers and the risk of developing MDD following a dysbiosis diagnosis. While the underlying pathophysiological mechanism remains unclear, the distinct microbiome variability between sexes necessitates further investigation.

### Regarding gender-specific microbiome diversity

Our review results are consistent with existing literature, emphasizing notable differences in the gut microbiota composition between individuals diagnosed with MDD and controls (9–18). These differences primarily involve microbial diversity and the prevalence of specific bacterial taxa. Four separate studies highlighted discernible variations in microbial diversity in both male and female MDD patients compared to their healthy counterparts (32–35). Notably, one study observed no significant difference in microbial diversity between male and female MDD patients (32). Most case-control studies found no alterations in alpha diversity among female MDD subjects compared to female healthy controls, while one study (35) reported reduced alpha diversity in female MDD subjects relative to healthy controls, mirroring a similar trend observed in male MDD subjects.

All studies examining beta diversity identified significant differences between female MDD patients and healthy controls (32–34), with two studies also noting distinct variations in beta diversity between male MDD patients and healthy controls (32, 33). One study focusing solely on females revealed alterations in beta diversity at the species level in female MDD subjects (34). Despite observing higher alpha diversity in healthy females compared to healthy males, this distinction was not observed in the depressed state (33).

These findings suggest gender-specific differences in the gut microbiome that may be influenced by various factors, such as the menstrual cycle stage, diet, age, and environmental factors. Overall, the results emphasize distinct beta diversity in both female and male MDD patients compared to healthy controls (32–34), with potential discrepancies in alpha diversity stemming from methodological variations in assessing microbiome diversity and the influence of confounding factors. Further clinical studies are warranted to comprehensively investigate the role of the gut microbiome in both male and female MDD patients, considering the potential implications for other diseases prevalent in females. The studies used various techniques, including 16S rRNA gene sequencing and shotgun metagenomic sequencing (SMG), to assess the microbiome. However, discrepancies in the methodologies employed suggest the need for standardized approaches in future research.

### In terms of gender-specific microbiome profiles

The current study reveals notable differences in the gut microbiome profiles of females with MDD in comparison to both healthy controls (HCs) and males with MDD. Analyzing data from four cross-sectional studies (32–35), we identified several differential abundances in bacterial clusters in both female and male MDD groups relative to HCs. These alterations primarily involved Actinobacteria, Bacteroidetes, Firmicutes, Proteobacteria, Fusobacteria, and Verrucomicrobia, which represent the dominant bacterial phyla in the human gut (29) Notably, despite previous literature suggesting Bacteroides as a signature gut microbe of MDD (17), our review unveiled inconsistent directions of compositional changes, which may be partly attributed to variations in the severity of depression. Hu et al. (35) also highlighted the influence of depression severity on gut microbiome alterations. Furthermore, a recent review on MDD and the gut microbiome by Knuesel and Mohajeri (22) identified disparities across studies, suggesting potential variations arising from different underlying causes and manifestations of depression across different age groups. Notably, the influence of confounding factors, such as the stage of the menstrual cycle, dietary patterns, physical activity, and environmental factors (28, 36) may contribute to the discrepancies observed in the findings. The current body of literature, however, lacks a sufficient number of studies investigating sex-specific differences in the gut microbiome concerning MDD.

### In the correlation of bacterial taxa with the severity of depressive symptoms

Several studies have indicated associations between specific bacterial taxa and the severity of depressive symptoms in individuals with MDD, as observed in the works of recent studies (19, 32–35). Notably, certain genera, including Anaerotruncus, Parabacteroides, and Anaeroglobus, were linked to increased depressive symptoms, whereas the presence of Clostridium XIVa, Erysipelotrichaceae incertae sedis, Streptococcus, Romboutsia, and Fusicatenibacter was associated with reduced symptoms. Despite Chen et al. (32) documenting correlations in males with MDD, the literature remains relatively limited and heterogeneous. A comprehensive review by Knuesel and Mohajeri (22) emphasized a negative correlation between Faecalibacterium and depressive symptoms, coupled with a positive correlation in cases of remission and improved quality of life. Similarly, Jiang et al. (9) demonstrated a negative association between Faecalibacterium prausnitzii (FP) and the severity of depressive symptoms. Likewise, Hu et al. (35) utilized shotgun sequencing, revealing a negative correlation between Faecalibacterium and depressive symptoms in a mixed-sex group of MDD patients with moderate depression. However, this correlation was not observed in the subgroup with severe depression, suggesting the potential confounding impact of depression severity. While the reviewed studies did not definitively establish the specific link between Faecalibacterium and the severity of depressive symptoms in females with MDD, they reported varying levels of Faecalibacterium in females with MDD compared to HCs. Despite existing disparities, Faecalibacterium remains a critical bacterial taxon of interest, previously associated with gut health and overall host well-being (37). Further exploration through improved methodological approaches, including controlling for sex as a biological factor and considering depression severity, is warranted to clarify the precise contribution of specific bacterial taxa to disease development or their status as a consequence of the disease.

### As a potential diagnostic microbial marker in depression

The evaluation of the diagnostic efficacy of microbial markers in females with MDD is still in its preliminary stages. Two separate studies have identified sex-specific gut microbial markers capable of distinguishing between males with MDD, females with MDD, and HCs (33, 34). Examination of how well these microbial signatures perform diagnostically showed that the area under the curve (AUC) values ranged from 0.79 to 0.92 for females and 0.79 for males diagnosed with MDD. Although these findings are limited due to sparse data and disparate methodologies, the identification of sex-specific microbial panels with potential diagnostic capabilities highlights the significance of sex stratification in MDD case-control studies. Additionally, this discovery provides crucial insights into the divergent pathophysiological mechanisms and prognostic variances between male and female MDD patients. Moreover, a study by Chung et al. (19) observed sex-specific disparities in the risk of developing MDD within five years following an initial dysbiosis diagnosis, with a notably stronger association among males compared to females. While specific microbial markers were not identified, this observation, in conjunction with existing evidence indicating the presence of sex-specific gut microbial profiles in MDD, emphasizes the potential for comprehensive characterization of sex-specific risk factors and the formulation of non-invasive gut microbial-based screening or diagnostic tools for MDD.

### The limitations of the present study

Include the heterogeneity in measurement and reporting methods, as well as the use of limited sample sizes and study designs, which impose certain restrictions on the interpretability of the results. However, these findings provide critical insights into the potential role of the gut microbiome in the context of MDD, especially concerning sex-specific differences. Future research should emphasize the inclusion of sex as a biological factor, conduct longitudinal studies to understand microbiome changes in response to clinical variations better, and carefully control for confounding factors to establish a more comprehensive understanding of the complex interplay between the gut microbiome and MDD.

## Conclusion

Despite the existing knowledge gaps and limitations, the findings underscore the significance of sex-specific differences in the gut microbiome of MDD patients. These insights hold important implications for potential advancements in the diagnosis, treatment, and understanding of the pathophysiology of MDD, emphasizing the necessity for further comprehensive investigations into the role of the gut microbiome in the context of sex-specific differences.

## Author contributions

LN: Conceptualization, Validation, Writing – review & editing, Data curation, Formal Analysis, Investigation, Methodology, Writing – original draft. GL: Validation, Writing – review & editing. SC: Validation, Writing – review & editing. MM: Validation, Writing – review & editing. AY: Validation, Writing – review & editing. BO: Conceptualization, Supervision, Validation, Writing – review & editing.
